# Pediatric Allergy and Immunology Practice During the COVID-19 Pandemic in Italy: Perspectives, Challenges, and Opportunities

**DOI:** 10.3389/fped.2020.565039

**Published:** 2020-11-23

**Authors:** Mattia Giovannini, Lorenzo Lodi, Lucrezia Sarti, Valentina Guarnieri, Simona Barni, Clementina Canessa, Giulia Liccioli, Giusi Mangone, Chiara Azzari, Francesca Mori, Silvia Ricci

**Affiliations:** ^1^Allergy Unit, Department of Pediatrics, Meyer Children's University Hospital, Florence, Italy; ^2^Department of Health Sciences, University of Florence, Florence, Italy; ^3^Immunology Unit, Department of Pediatrics, Meyer Children's University Hospital, Florence, Italy

**Keywords:** allergy, immunology, clinical practice, COVID-19, pediatrics

## Introduction

The coronavirus disease 2019 (COVID-19) pandemic is one of the most complex issues for healthcare systems worldwide, accounting for 269,042 cases and 35,957 associated deaths up to September 1, 2020, in Italy. Approximately 4% of the patients belonged to the pediatric population, and only four fatalities were recorded in this age group (1). Moreover, it was demonstrated that in children, COVID-19 has milder clinical manifestations and a less severe clinical course (2–5). However, as pointed out by Malipiero et al. (6), the necessity to limit potential infections, together with striving to deliver standard high-quality care, was a huge challenge to face. To the best of our knowledge, no specific pediatric experiences have been described in the scientific literature. For this reason, we aim to share ours at Meyer Children's University Hospital in Florence.

General measures were adopted by our institution for preventing potential severe acute respiratory syndrome coronavirus 2 (SARS-CoV-2) nosocomial infections. Mass temperature body screening of pediatric patients, parents, and healthcare workers was performed routinely at the entrance of the hospital with a thermal scanner. Furthermore, all the healthcare personnel underwent SARS-CoV-2 infection screening through real-time polymerase chain reaction on a nasopharyngeal swab and serological test; patients and their parents underwent the same investigations if deemed appropriate. Inside the hospital, the use of a protective mask and frequent handwashing or sanitization was considered mandatory.

## Our Experience

Recent guidelines (7–12) regarding practice during the COVID-19 pandemic were followed to manage the referrals and scheduled clinical activity decreased. However, from February 28, 2020, general pediatricians (GPs) gave their availability to be 12 hours/7 days a week on call. They were persistently in contact with other hospital service resources, representing a strategic collaborative resource for limiting access to patients with no longer deferrable clinical needs. In addition, the Allergy and Immunology Units were continuously in touch with patients in follow-up.

The Allergy Unit granted face-to-face access to patients to perform a first clinical evaluation in case of severe allergic reactions (e.g., anaphylaxis), rhinoconjunctivitis without response to topical treatments, uncontrolled asthma, administration of Hymenoptera venom immunotherapy, and biologic treatments (if not possible at home or by a local center). Molecular testing on nasopharyngeal swab ruled out the SARS-CoV-2 infection for all cases where an acute infection was suspected before they accessed our unit. Specific healthcare personnel performed the test in a dedicated area of the hospital.

The Immunology Unit granted face-to-face access to patients with high suspicion of severe combined immunodeficiency (SCID) or profound combined immunodeficiency. Newborn screening for SCID, based on T-cell receptor excision circles and κ-deleting recombination receptor excision circles assays, was ensured during the entire period, and positive patients were recalled for clinical evaluation. Furthermore, we continued to guarantee in-person clinical evaluations for all chronic primary immunodeficiency (PID) patients with acute clinical conditions. In fact, subjects who presented with uncontrolled autoinflammation phenomena, autoimmune exacerbations, or signs or symptoms for proliferative disease development or clinical manifestations of suspected invasive infections were promptly evaluated at our unit, as these clinical conditions were considered undelayable. For all cases where an acute infection was suspected, the SARS-CoV-2 infection was ruled out by molecular testing on nasopharyngeal swab before they accessed our Immunology Unit. The test was performed by specific healthcare personnel in a dedicated area of the hospital. Serology tests were not used for PID patients, as recommended. Moreover, we invited all PID patients with ongoing immunoglobulin replacement therapies to switch to home treatments through subcutaneous infusions. However, in a minority of cases, it was not possible to transit to home therapy. In such cases, as well as for patients who were receiving intravenous immunosuppressive therapy, access to the Immunology Unit was regularly allowed, as they were considered urgent therapies.

Allergy and Immunology Units provide immunization services for children who require the administration of vaccines in a hospital setting. Efforts were adopted to adhere to the regular immunization schedule to avoid delays that may have caused an increased risk of vaccine-preventable diseases.

Telemedicine, through a remote medical and nursing support with video consultations and digital educational materials, was available to GPs and all patients according to their needs. Indeed, as shown in the literature, telemedicine proved to be an effective, feasible, and appreciated tool during the COVID-19 pandemic (13–15).

To minimize the risk of infections, specialists, residents, and nurses organized weekly shifts inside the units, staying home in turn. This unique situation determined the need for a high degree of flexibility by the staff.

The Laboratory of Immunology and Molecular Microbiology was under high pressure due to the large number of requests linked both to COVID-19 diagnosis in children and SARS-CoV-2 infection hospital surveillance. For this reason, even clinical staff made themselves available, based on their individual personal skills, to support these activities.

On the academic side, recruiting for clinical trials was temporarily stopped to ensure the safety of the participants. All research efforts were directed to support the scientific community in the fight against COVID-19. Teaching activity was provided through teleconference as well as exams.

## Conclusion

Allergy and immunology practice during the COVID-19 pandemic represented a huge challenge to face. At the same time, this period represented a unique opportunity for us to learn several lessons. Telemedicine may play a more and more central role in managing allergic and immunological deferrable clinical needs in the future. For this reason, it seems reasonable to foster investments and technological advancements in order to expand this field. In addition, the necessity of organizing and focusing health resources made us realize the need to think out of the box regarding the prioritization of care in different clinical situations. We were obliged to get out of our comfort zone and change our perspectives, discovering new individual attitudes and coping resources. Nonetheless, it would not have been possible to deal with this difficult scenario without working together as a coordinated team, which represented the cornerstone of our experience.

## Author Contributions

MG and CA conceptualized the work. MG, VG, FM, and SR have drafted the manuscript. MG, LL, LS, VG, SB, CC, GL, GM, CA, FM, and SR performed the investigations and critically revised the manuscript. All authors approved the final version of the manuscript as submitted and agreed to be accountable for all aspects of the work.

## Conflict of Interest

The authors declare that the research was conducted in the absence of any commercial or financial relationships that could be construed as a potential conflict of interest.

## References

[1] Task force COVID-19 del Dipartimento Malattie Infettive e Servizio di Informatica. In: Epidemia COVID-19, Aggiornamento Nazionale: 1 Settembre 2020. Istituto Superiore di Sanità. Available online at: https://www.epicentro.iss.it/coronavirus/bollettino/Bollettino-sorveglianza-integrata-COVID-19_1-settembre-2020.pdf (accessed September 10, 2020).

[2] Lu X Zhang L Du H Zhang J Li YY Qu J. SARS-CoV-2 infection in children. N Engl J Med. (2020) 382:1663–5. 10.1056/NEJMc2005073 32187458 PMC7121177

[3] Castagnoli R Votto M Licari A Brambilla I Bruno R Perlini S. Severe acute respiratory syndrome coronavirus 2 (SARS-CoV-2) infection in children and adolescents. JAMA Pediatr. (2020) 174:882–9. 10.1001/jamapediatrics.2020.1467 32320004

[4] Riggioni C Comberiati P Giovannini M Agache I Akdis M Alves-Correia M. A compendium answering 150 questions on COVID-19 and SARS-CoV-2. Allergy. (2020). [Epub ahead of print]. 10.1111/all.14449 32535955 PMC7323196

[5] Garazzino S Montagnani C Donà D Meini A Felici E Vergine G. Multicentre Italian study of SARS-CoV-2 infection in children and adolescents, preliminary data as at 10 April 2020. Eurosurveillance. (2020) 25:2000600. 10.2807/1560-7917.ES.2020.25.18.2000600 32400362 PMC7219028

[6] Malipiero G Paoletti G Puggioni F Racca F Ferri S Marsala A. An academic allergy unit during COVID-19 pandemic in Italy. J Allergy Clin Immunol. (2020) 146:227. 10.1016/j.jaci.2020.04.003 32317114 PMC7161527

[7] Shaker MS Oppenheimer J Grayson M Stukus D Hartog N Hsieh EWY. COVID-19: pandemic contingency planning for the allergy and immunology clinic. J Allergy Clin Immunol Pract. (2020) 8:1477–88.e5. 10.1016/j.jaip.2020.03.012 32224232 PMC7195089

[8] Brough HA Kalayci O Sediva A Untersmayr E Munblit D Rodriquez del Rio P. Managing childhood allergies and immunodeficiencies during respiratory virus epidemics – the 2020 COVID-19 pandemic. Pediatr Allergy Immunol. (2020). [Epub ahead of print]. 10.1111/pai.13262 32319129 PMC7264548

[9] IPOPI, ESID, INGID, APSID, ARAPID, ASID, CIS, LASID, SEAPID Joint Statement on the Current Epidemics of New Coronavirus SARS-CoV-2 — COVID-19. Available online at: https://ipopi.org/wp-content/uploads/2020/03/COVID19_Mar13v1_EN.pdf (accessed May 23, 2020).

[10] World Health Organization Immunization in the Context of COVID-19 Pandemic. Available online at: https://apps.who.int/iris/bitstream/handle/10665/331818/WHO-2019-nCoV-immunization_services-FAQ-2020.1-eng.pdf?sequence=1&isAllowed=y (accessed May 23, 2020).

[11] Pfaar O Klimek L Jutel M Akdis C Bousquet J Breiteneder H. COVID-19 pandemic: practical considerations on the organization of an allergy clinic – an EAACI/ARIA position paper. Allergy. (2020). [Epub ahead of print]. 10.1111/all.14453 32531110 PMC7323448

[12] Cardinale F Ciprandi G Barberi S Bernardini R Caffarelli C Calvani M. Consensus statement of the Italian society of pediatric allergy and immunology for the pragmatic management of children and adolescents with allergic or immunological diseases during the COVID-19 pandemic. Ital J Pediatr. (2020) 46:84. 10.1186/s13052-020-00843-2 32546234 PMC7296524

[13] Codispoti CD Bandi S Moy JN Mahdavinia M. Running a virtual allergy division and training program in the time of COVID-19 pandemic. J Allergy Clin Immunol. (2020) 145:1357–9. 10.1016/j.jaci.2020.03.018 32243877 PMC7201123

[14] Keswani A Brooks JP Khoury P The future of telehealth in allergy and immunology training. J Allergy Clin Immunol Pract. (2020) 8:2135–41. 10.1016/j.jaip.2020.05.009 32426217 PMC7233253

[15] Portnoy J Waller M Elliott T Telemedicine in the era of COVID-19. J Allergy Clin Immunol Pract. (2020) 8:1489–91. 10.1016/j.jaip.2020.03.008 32220575 PMC7104202

